# Experimental evidence for flexibility in provisioning behavior in a cooperatively breeding bird

**DOI:** 10.1093/beheco/arag021

**Published:** 2026-02-26

**Authors:** María Espinosa-Colín, Carlos de la Cruz, Jorge S Gutiérrez

**Affiliations:** Department of Anatomy, Cell Biology and Zoology, Faculty of Sciences, University of Extremadura, Badajoz 06006, Spain; Department of Anatomy, Cell Biology and Zoology, Faculty of Sciences, University of Extremadura, Badajoz 06006, Spain; Department of Anatomy, Cell Biology and Zoology, Faculty of Sciences, University of Extremadura, Badajoz 06006, Spain; Ecology in the Anthropocene, Associated Unit CSIC-UEX, Faculty of Sciences, University of Extremadura, Badajoz 06006, Spain

**Keywords:** brood manipulation, cooperative breeding, corvid, nestling period, parent–offspring conflict, parental care

## Abstract

The flexible-investment hypothesis posits that parents should allocate their resources according to offspring needs. The available evidence suggests that this is valid in most avian biparental care systems; yet, experiments designed to quantify the degree to which caregivers respond to the demands of offspring are rare in cooperative breeding systems, where offspring are reared by individuals additional to the breeding pair (ie helpers). By cross-fostering Iberian magpie (*Cyanopica cooki*) chicks of different ages, we tested whether breeders and helpers adjust both the duration and intensity of care in response to brood developmental stage. Both breeders and helpers (all males) prolonged their nestling care period when raising chicks younger than their own and shortened it when raising chicks older than their own, with no detectable effect on reproductive success. Overall, provisioning increased when the chick-rearing period was experimentally shortened and decreased when it was prolonged. Yet, the magnitude of these responses differed among carer statuses. In the shortening experiment, female breeders showed the strongest increase in provisioning, whereas helpers showed little or no change. In contrast, during brood prolongation, provisioning declined in all carer types but was most pronounced in breeders. Overall, these results support the flexible-investment hypothesis while indicating that helpers exhibit more limited flexibility in their feeding responses than breeders. Combined with previous observational work, our results are consistent with the idea that helping behavior may function in part as a signal to gain direct benefits through reciprocity.

## Teaser

I had uploaded the lay summary during the submission process: In cooperative animals, caregivers are expected to adjust their effort for maximum benefit. By swapping chicks of different ages between nests in Iberian magpies, we found that parents and helpers adjusted both care duration and feeding — more for younger chicks, less for older — without reducing reproductive success. Helpers appeared less flexible than parents, suggesting their role may be more about signaling commitment than optimizing care.

## Introduction

Parental care, defined as any form of parental behavior that increases the growth or survival of offspring, is common and taxonomically widespread across the animal kingdom (Clutton-Brock and Scott 1991; Royle et al. 2012). Familiar examples include the construction of nests and burrows, protection of offspring from predators and parasites, and feeding of offspring (provisioning) before and after birth or hatching (Clutton-Brock and Scott 1991; Royle et al. 2012, 2014). Although parental care benefits parents by increasing offspring survival and increasing their reproductive success, it is costly in terms of time, energy, or ability to raise other offspring (Royle et al. 2014). From an evolutionary point of view, parental care is therefore expected to be favored when the benefits to parents outweigh the costs and when environmental conditions are harsh and/or unpredictable (Wilson 1975; Gonzalez-Voyer and Kolm 2010; Meunier and Kölliker 2012; Moss and Moore 2021). Such conditions are met by many birds and mammals, which produce offspring that require substantial postnatal care to survive, so the benefits of care generally outweigh the costs. This is particularly evident in altricial species, where offspring hatch or are born at a markedly early stage of development. Nevertheless, both intrinsic (such as social structure) and extrinsic factors (such as environmental conditions) determine the benefits and costs associated with parental care and thus parent–offspring conflicts over the amount and duration of parental care (Trivers 1974; Meunier and Kölliker 2012; Royle et al. 2012, 2014).

The ecology and evolution of flexible parenting have been the focus of considerable debate (Royle et al. 2014; Moss and Moore 2021), with much of the empirical evidence for and against flexibility of parenting behaviors coming from studies of birds. Many studies have experimentally manipulated brood size or age to test whether parents respond to proximate cues of nestling demand (Wright and Dingemanse 1999; Erikstad et al. 2009; Harding et al. 2009; Koenig and Walters 2012; Rehling et al. 2012; Soler et al. 2013; Gow and Wiebe 2014; Pelletier et al. 2016; Khwaja et al. 2017; White and Dawson 2022). The results have been mixed: while parents of most species adjust their investment (feeding rates, foraging strategy, or duration of parental care) when offspring demand is manipulated, others show limited or no parental flexibility (reviewed by Erikstad et al. [2009]; Gow and Wiebe [2014]). For example, in a cross-fostering experiment, zebra finch (*Taeniopygia guttata*) parents provisioned nestlings for longer when they received younger foster chicks and for less time when they received older foster chicks (Rehling et al. 2012). Likewise, magpies (*Pica pica*) parents adjusted the duration of care according to the requirements of cross-fostered nestlings (Soler et al. 2013). Interestingly, these studies suggest that the duration of parental care is influenced by the state of development of offspring, underscoring the biological importance of an optimal parental care (Trivers 1974; Verboven and Tinbergen 2002). In contrast, parents of several seabird species provide care for a fixed time period (but see Erikstad et al. [2009]; Harding et al. [2009]). Parents may also have the capacity to adjust feeding rate and the type of prey they provide to offspring demand over just a few visits (Pelletier et al. 2016; White and Dawson 2022). Generally, provisioning rates are lower when broods are reduced in size or age and greater when broods were enlarged or prolonged (Gow and Wiebe 2014; Pelletier et al. 2016). It has been hypothesized that in long-lived species with large home ranges and extended nestling periods parents encounter energetic constraints that limit their capacity to respond to enlarged or prolonged broods (Erikstad et al. 2009; Gow and Wiebe 2014). This raises the issue of the interplay of life history and the ecological conditions in determining amount of care provided by parents.

The available evidence suggests that the flexible-investment hypothesis is valid in most biparental care systems; yet, experiments designed to quantify the degree to which caregivers respond to the demands of offspring are rare in cooperative breeding systems, where offspring are reared by individuals additional to the breeding pair (Wright and Dingemanse 1999; Nam et al. 2011; Koenig and Walters 2012). Indeed, parental care and investment may be particularly variable in cooperative breeders, as asymmetries in relatedness among family members may lead to genetic conflicts over the amount and duration of parental care (Royle et al. 2012). In many cooperative species, young (naïve) helpers are less likely to help than older (experienced) ones, which suggests a higher cost of care for juveniles that lack mature foraging and/or provisioning skills (eg Woxvold et al. 2006; Klauke et al. 2014). Understanding the provisioning behavior of carers in relation to age can yield important insight into how allocation rules are acquired over time (Klauke et al. 2014).

In cooperatively breeding acorn woodpeckers (*Melanerpes formicivorus*), both breeders and helpers adjust their feeding rate to maintain about the same per-nestling feeding rate (Koenig and Walters 2012). However, breeders but not helpers decrease their feeding rate in response to decreased brood size, suggesting that the “feeding rules” of helpers are less flexible than those of breeders. This implies that helpers are less able to assess the needs of nestlings than breeders, or that breeders coerce helpers to sustain their feeding rate even when the brood size is reduced (Koenig and Walters 2012). On the other hand, both parents and helpers compensate for experimental changes in the provisioning effort of others in Arabian babblers (*Turdoides squamiceps*) (Wright and Dingemanse 1999).

Here, we examined the flexibility (or lack thereof) in the duration of parental care and rate of chick provisioning in the cooperatively breeding Iberian magpie (*Cyanopica cooki*). We achieved this by cross-fostering chicks of different ages between nests, thereby potentially prolonging and shortening the chick-rearing period. Furthermore, we investigated duration of parental care relates to chick development and reproductive success. In our study system, approximately half of the breeding pairs have helpers at the nest (usually 1 to 3 helpers per nest), which assist in protecting and feeding offspring (de la Cruz et al. 2019). Breeders and helpers respond differently to nestling demand, with breeders increasing their provisioning effort as brood demand rises, while helper contributions vary according to their relatedness to the breeders. That is, helpers related to the parents (typically juveniles) deliver larger food loads on each trip, consistent with the higher feeding rates observed in kin compared with nonkin helpers (Valencia et al. 2003, 2006a; de la Cruz et al. 2019). Based on these observational studies in Iberian magpies and previous literature on the flexible-investment hypothesis (see above), we hypothesized that caregivers should respond to brood demand according to the breeding status and age of the carer. We first predicted that both parents and helpers would provision chicks for longer in prolonged nests and for less time in shortened ones. We then predicted that helpers would not respond as much as parents to the increase or decrease of brood demand. Finally, we predicted that less experienced young birds would feed less than adult ones.

## Methods

### Ethical note

All procedures followed ASAB/ABS guidelines (ASAB Ethical Committee/ABS Animal Care Committee 2023) and Spanish regulations for animal behavioral research and were approved by the Ethics Committee of the University of Extremadura and the Government of Extremadura (CN0013/19/ANN and CN0014/18/AAN). To create experimental broods, we cross-fostered Iberian magpie nestlings by carefully transporting them in an artificial cotton nest. Transfer of chicks between nests took less than 15 min, and all of them were accepted by the foster parents. No chick died during transportation or after the manipulation, and none of the nests from which chicks were taken or introduced was deserted.

### Study population

**Figure 1:**
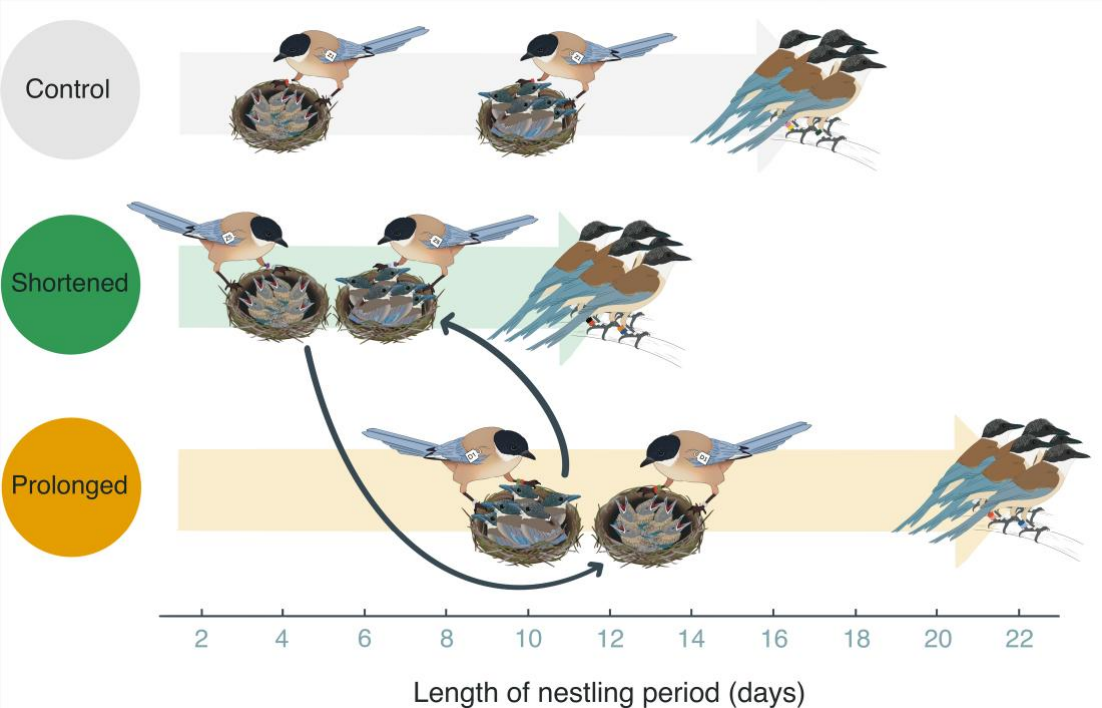
Schematic presentation of the experimental approach. The experimental swapping of chicks of different ages (4 or 10 d) resulted in nestling periods that were either shortened or prolonged in comparison to control chicks. Thick gray arrows show the length of chick-rearing periods and thin black arrow show the swapping of chicks. The swapping caused a mismatch between feeding rates and chick needs.

The Iberian magpie is a cooperative corvid endemic to the Iberian Peninsula (de la Cruz and Valencia 2016). Iberian magpies breed in a highly flexible cooperative system, in which some individuals help at different stages of the breeding cycle (from nest building to feeding the incubating female and young; de la Cruz et al. 2019). Helpers are almost exclusively males, either adults or juveniles (de la Cruz et al. 2019). Moreover, there are two types of helpers: first option helpers are typically juveniles that do not attempt their own reproduction and help one or both parents; whereas second option helpers are failed breeders that switch from breeding to helping at nests of nonkin in the proximities (Valencia et al. 2003). On average, half of the hatched nests are assisted by helpers, and nest success is positively related to the presence of helpers (Valencia et al. 2003). This population is part of a long-term study in Badajoz, southwestern Spain (39°03′N, 6°48′W). The study area consists of an open holm oak (*Quercus ilex*) woodland used as a pastureland for cattle (for more details, see Valencia 2000; Valencia et al. 2003; de la Cruz and Valencia 2016; de la Cruz et al. 2022). All individuals in the study were color-ringed with a unique combination of one numbered metal ring, two colored plastic rings, and a patagial wing tags (Fig. 1). Thus, all birds in the present study and their family histories were known.

### Experimental procedure

We performed this experiment during the 1998, 1999, and 2018 breeding seasons (from March to July). At the beginning of the breeding season, the study area was prospected to locate nests within the colonies. Every nest was either directly observed or video recorded to identify breeder and helper individuals. Helper contribution is rare during the incubation phase, so when a bird was repeatedly observed feeding a female at the nest it was identified as the breeding male. In this species, only the female incubates the clutch. On the other hand, birds observed occasionally at the nest during this phase or those that were only present during the brooding phase were considered helpers (eg, de la Cruz et al. 2019, 2022). As expected, all helpers were males. Nests were checked nearly every day until nestlings hatched to determine nestling age correctly. At hatching, nestlings were marked by painting a specific toenail to allow individual recognition and then ringed with metal rings when 12 days old (Valencia et al. 2006b).

Two days after hatching, broods were assigned randomly to three groups: control broods, prolonged broods, and shortened broods. The treatment involved manipulation of the age of nestlings that parents (aided or not by helpers) provisioned by swapping entire broods of similar size (we tolerated a brood size difference of 1 chick between swapped broods; mean brood size±SE was 5.84 ± 0.09 chicks for shortened broods and 5.57 ± 0.11 chicks for prolonged broods) but of different ages between nests (4- to 5-day-old chicks vs. 10- to 11-day-old chicks). Unaltered broods (5.07 ± 0.16 chicks) served as controls; they received the same number of visits and level of handling (including toenail marking) as nests of the experimental groups except for the cross-fostering. Control broods were assigned such that the age of nestlings was comparable to that of experimental broods; hence why controls were divided into two groups (“control-shortened” and “control-prolonged”). In total, we had 28 experimental nests (14 shortened and 14 prolonged, of which 6 and 4 had helpers, respectively) and 43 control nests (of which 11 had helpers, respectively).

**Figure 2:**
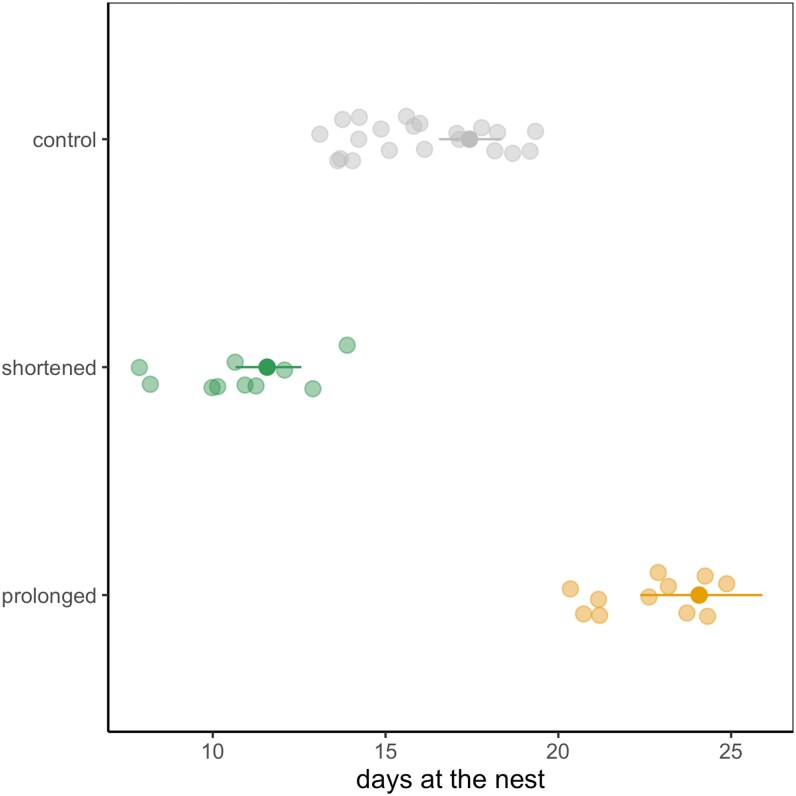
Predicted number of days at control (unaltered) or experimental (foster) nests from a GLM with a Conway–Maxwell–Poisson distribution. Predicted values (solid points with 95% CI lines) are based on fixed-effect estimates from the model, adjusted to: help = 0, laying date = 19.61, fledglings = 4, year = 1998. Raw data points (jittered) are uncorrected and represent individual observations. Model estimates account for other covariates in the model and may therefore differ from the raw data. Note that foster nests had their own control nests to account for the confounding effect of time when swapping chicks.

To examine whether parents and helpers compensate for experimental changes in the provisioning effort, we conducted observations before and after the swap throughout the rearing period. Each day, we conducted nearly simultaneous 1-h feeding observations to calculate hourly feeding rate (number of times each individual visited the nest to feed the chicks) (de la Cruz et al. 2019, 2022). As the birds may avoid the hottest hours of the day for feeding activities, all the observations were carried out in the morning. We focused on the 2 days before and after the swap for both shortened broods (days 3 to 4 and 5 to 6, respectively) and prolonged broods (days 9 to 10 and 11 to 12, respectively). For control broods, we selected the same days to be compared with the respective experimental broods and thus make observations fully comparable. We made this choice to account for the confounding effect of time when swapping chicks (Erikstad et al. 2009). For instance, chicks from shortened nests fledged about 10 days earlier than those from prolonged ones, thus preventing comparisons between shortened and control broods after day 12 (Fig. 2). Hence why control broods were compared with shortened and prolonged broods separately and are shown as “control-shortened” and “control-prolonged” in the provisioning analyses, respectively (see below). We collected a total of 1,141 hourly feeding rates (ie, hours of observation) throughout the rearing period, of which 601 came from the days of interest (± 2 days after the swap) and were used in the provisioning analyses. Experimental treatments were balanced across years (see Gutiérrez et al. 2026).For a schematic presentation of the experimental approach, see Fig. 1.

In addition to feeding visit rate, we recorded the number of days since laying/hatching, number of chicks present in the nest, and number of fledglings produced. To assess potential effects of treatment on nestling development, one of us measured nestling body mass (± 0.1 g), tarsus length (± 0.01 mm), and flattened wing chord (± 1 mm) at 12 days posthatching. Nestlings were measured on day 12 rather than at later ages to prevent premature fledging (nestling period c. 14 to 16 days; de la Cruz et al. 2019). Because the first 3 to 4 eggs hatch synchronously and the remaining eggs hatch at irregular intervals (de la Cruz and Valencia 2016), thus making difficult to accurately determine the hatching date of older nestlings, we only analyzed data from the three older chicks within a given brood to reduce variability in size caused by differences in age (*N* = 98 nestlings: 28 from 10 shortened broods; 33 from 12 prolonged broods; 12 from 5 control-shortened broods; and 25 from 9 control-prolonged broods). We also analyzed the effect of the treatment on nestling body condition using the scaled mass index, a method that standardizes body mass to a fixed body size (Peig and Green 2009). The fixed value of body size was the mean value of tarsus length on day 12 for the study population: 31.32 ± 1.82 mm (*N* = 505 nestlings).

### Statistical analysis

To assess our hypotheses, we constructed a set of linear and generalized linear (mixed) models (LMM and GLMM) using the R environment version 4.2.1 (R Core Team 2022).

To test whether shortened, prolonged, and control nests had a similar brood size (before the swapping) and reproductive success (after the swapping), we first fitted separate Poisson GLMM with the number of chicks or fledglings as the response variables, laying date (days after 1 April) as a covariate, and group (shortened, prolonged, control-shortened, control-prolonged), and year as fixed factors using the package “lme4” (Bates et al. 2015). Unlike the provisioning and morphometric models described below, we did not include individual or nest identity as random effects, as the brood size data were analyzed at the nest level (ie, using a fixed value per nest). The brood size model was underdispersed and a simulation of scaled model residuals using the package “DHARMa” (Hartig 2024) indicated significant deviation of model residuals (Kolmogorov-Smirnov test: (*D* = 0.367, *P* < 0.001). Thus, we fitted a GLMM with a Conway–Maxwell Poisson (family compois) distribution using the package “glmmTMB” (Brooks et al. 2017), which can account for underdispersion or overdispersion commonly encountered in count data (Shmueli et al. 2005); this model fitted the data adequately (see Results). In contrast, the fledgling model showed overdispersion and significant deviation of model residuals (*D* = 1.962, *P* < 0.001). Again, a Conway–Maxwell Poisson distribution model yielded a good fit to the data (see Results).

To test for differences in parental care period between treatments, we fitted a GLM with a Poisson error distribution analyzing the length of nestling period (ie, days at the foster or control nest; all chicks fledged on the same day). We included group, helping (yes/no), and year as fixed factors, and laying date and brood size as covariates. This model was underdispersed and showed significant deviation of model residuals (*D* = 0.116, *P* = 0.001); however, a Conway–Maxwell–Poisson distribution model with the number of days at the foster/control nest as the response variable fitted the data adequately (see Results). We then analyzed differences among treatments by conducting multiple post hoc comparisons using the package “multcomp” (Hothorn et al. 2008). Although none of the nests were deserted, some were predated a few days after swapping and then excluded from this analysis (3 out of 14 “shortened” nests and 4 out of 14 “prolonged” nests, and 20 out of 43 control nests); therefore, sample sizes differ from the provisioning analyses (see below). Predation rates were similar among control, prolonged, and shortened nests (Fisher's exact test, *P* = 0.205). The observed predation rates in our experimental and control nests are consistent with natural levels in the study population, where nest predation can affects over 50% of reproductive attempts annually and represents the primary cause of reproductive failure (Avilés et al. 2025).

To examine the effect of the experimental manipulation on chick provisioning and potential differences between statuses, we fitted GLMMs with a Poisson response distribution and log link function: one testing the effect of prolonging the chick-rearing period and the other testing the effect of shortening it. We opted to analyze these treatments separately because swapping chicks of differing ages confounds treatments with seasonal effects (Erikstad et al. 2009). In both models, we included hourly feeding rate (at the individual scale) as our response variable, group (experimental vs. control), period (before or after the swap), status (female breeder, male breeder, or helper), age (1-year old or older, there after “juvenile” or “adult,” respectively) and year as fixed factors, and day since hatching as a covariate. We included the interactions group × period and status × period, as well as the three-way interaction group × status × period to assess provisioning flexibility. In the provisioning models, we also included individual and nest identity as random effects to account for the nonindependence of observations from nestlings from the same nests. To further explore significant interactions, we conducted post hoc pairwise comparisons based on estimated marginal means using the package “emmeans” (Lenth 2025). First, we assessed within-status flexibility by comparing provisioning rates before and after the manipulation separately for each combination of carer status and experimental group. These contrasts allowed us to quantify the change in provisioning associated with the manipulation within each status. Second, to assess differences in flexibility among statuses, we compared the magnitude of these before–after changes between statuses within each experimental group. All contrasts were performed on the response scale and are reported as rate ratios.

To assess differences in body mass, wing length, tarsus length, and body condition of nestlings just before fledging (response variables), we fitted LMMs with the experimental treatment, helping, and year as fixed factors, as well as date and brood size as covariables. We included nest identity as a random factor to account for the nonindependence of nestlings from the same nest.

We checked the fit of all models through visual examination of residuals and using the packages “DHARMa” (Hartig 2024) and “performance” (Lüdecke et al. 2021). We also discarded collinearity (variance inflation factors < 3) using the “car” package (Fox and Weisberg 2019). Model diagnostics indicated no deviations from the model assumptions. To assess the goodness-of-fit, we determined the conditional coefficient of determination (*R*^2^c, variance explained by both fixed and random factors (Nakagawa and Schielzeth 2013) using the “MuMIn” package (Bartoń 2022). For data visualization, we used the “ggplot2” package (Wickham 2016).

## Results

### Experimental design and parental care period

At the time of manipulation, brood size was similar between shortened (raw mean ± standard error: 5.86 ± 0.234) and prolonged (5.43 ± 0.416) nests (Table 1), as well as between experimental and control (4.96 ± 0.234) nests (*P* > 0.3 in all post hoc comparisons). Moreover, brood size was negatively influenced by laying date but did not vary between years (Table 1; *P* > 0.5 in post hoc all-pair comparisons).

**Table 1:** Models explaining variation in (a) brood size (at the time of the manipulation) and (b) reproductive success (after the manipulation) in shortened, prolonged, and control nests.

Model	Estimate	SE	*Z*	*P*
(a) Brood size				
Intercept	1.739	0.073	23.763	<0.001
Shortened nests	0.114	0.084	1.357	0.175
Prolonged nests	0.003	0.089	0.032	0.975
**Std. laying date**	**−0**.**007**	**0**.**002**	**−2**.**831**	**0**.**005**
Year 1999	0.011	0.075	0.146	0.884
Year 2018	0.099	0.101	0.9585	0.325
(b) Reproductive success				
Intercept	0.921	0.274	3.354	0.001
Shortened nests	0.322	0.304	1.057	0.291
Prolonged nests	0.279	0.317	0.883	0.377
Std. laying date	−0.016	0.009	−1733	0.083
Year 1999	0.191	0.270	0.705	0.481
Year 2018	0.286	0.381	0.752	0.452

Reference levels for group and year are “control” and “1998,” respectively. Significant explanatory variables are in bold. See Methods for details.

As expected, the duration of the nestling period was longer in prolonged broods (raw mean: 22.6 ± 1.63 days) and shorter in shortened broods (10.8 ± 1.93 days), compared with control ones (16.0 ± 1.95 days) (Fig. 2). In the Conway–Maxwell Poisson GLMM, treatment and year emerged as significant predictors of nestling period duration (*P* < 0.001 in both cases). Parents from the prolonged treatment cared for their foster brood for longer (estimate = 0.323, 95% CIs = 0.255 to 0.390), whereas those from the shortened treatment cared for their foster brood for less time (estimate = −0.409, 95% CIs = −0.488 to −0.330) compared with those from their respective controls (Fig. 2). Indeed, post hoc tests showed that the duration of the nestling period differed between experimental and control nests (always *P* < 0.001). Duration was slightly shorter in year 1999 than in 1998 and 2018, albeit the year effect was small (estimate = −0.163 ± 0.031, *P* < 0.001).

None of the nests were deserted, and reproductive success did not vary among treatments (*P* > 0.5 in all post hoc comparisons; Table 1).

### Effect of shortening on chick provisioning

**Figure 3:**
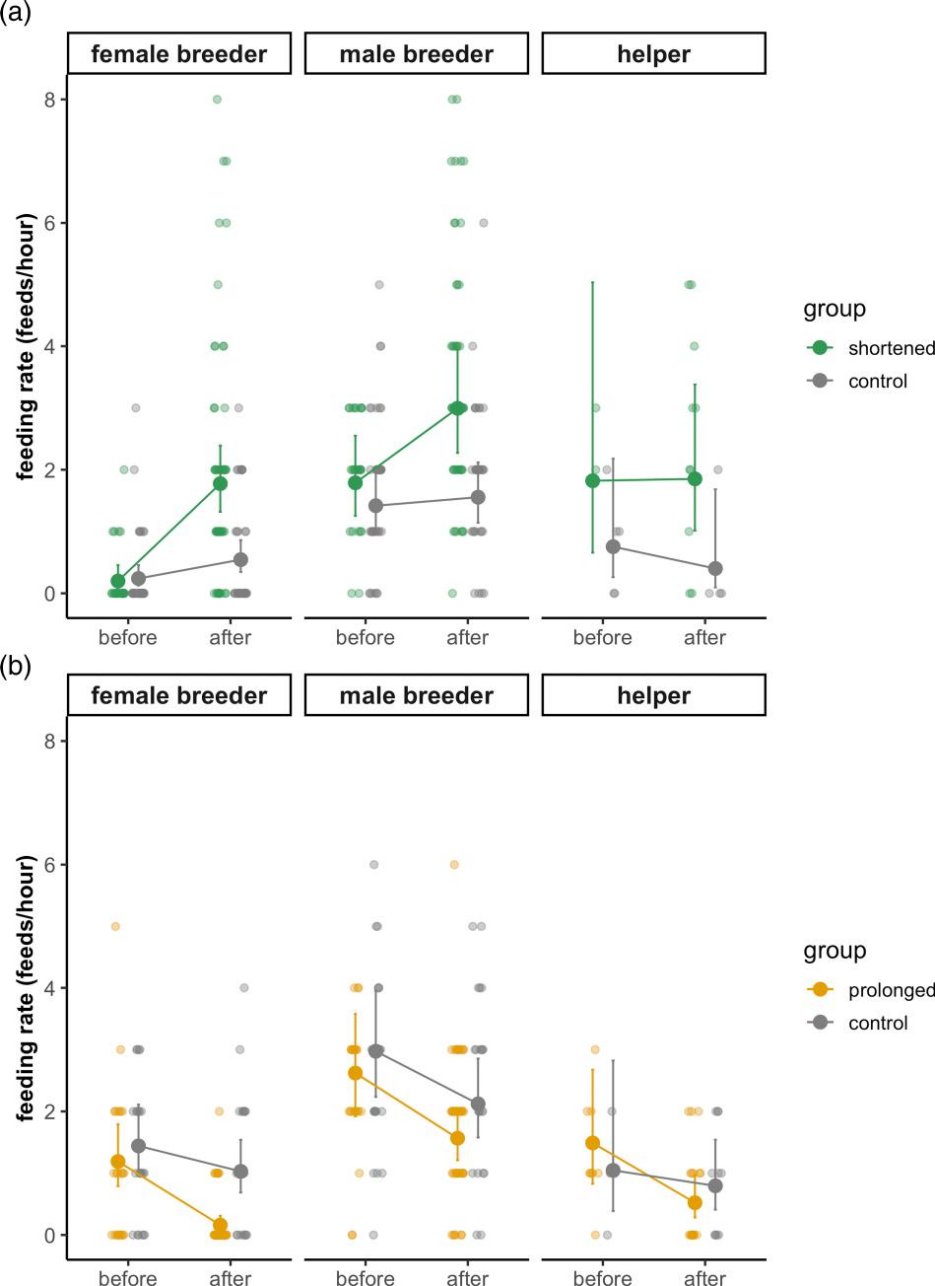
Effect on the provisioning rate of a) shortening and b) prolonging the brood-rearing period. Dots are predicted estimates from separate GLMM with Poisson distributions, and vertical lines are the 95% confidence intervals based on fixed-effect uncertainty. Predicted values (solid points with 95% CI lines) are based on fixed-effect estimates from the model, adjusted to help = 0, laying date = 19.61, fledglings = 4, year = 1998, and nest = 0 (population-level). Raw data points (jittered) are uncorrected and represent individual observations. Model estimates account for other covariates in the model and may therefore differ from the raw data.

Shortening the chick-rearing period was associated with a general increase in provisioning rates; however, the magnitude of this increase differed among carer statuses (group × status × period interaction; Table 2, Fig. 3). In experimentally shortened nests, female breeders showed a strong increase in provisioning after receiving older chicks, feeding at rates nearly nine times higher than before the manipulation (within-status post hoc comparisons: ratio = 8.915 ± 3.776, *P* < 0.001; Table 3; Fig. 3). Male breeders also increased provisioning, although to a lesser extent (ratio = 1.674 ± 0.290, *P* = 0.003; Table 3, Fig. 3). In contrast, helpers showed no detectable change in provisioning following the manipulation (ratio = 1.017 ± 0.514, *P* = 0.974; Table 3, Fig. 3). In control nests, female breeders showed a moderate increase in feeding rates (ratio = 2.273 ± 0.872, *P* = 0.032; Table 3, Fig. 3), whereas neither male breeders (ratio = 1.098 ± 0.220, *P* = 0.642; Table 3) nor helpers (ratio = 0.532 ± 0.472, *P* = 0.477; Table 3, Fig. 3) exhibited significant changes.

**Table 2:** Mixed models explaining variation provisioning rates after (a) shortening and (b) prolonging the chick-rearing period.

Model	Estimate	SE	*Z*	*P*
(a) Shortening				
Intercept	−1.528	0.479	−3.190	0.001
Group_control	−0.186	0.521	0.357	0.721
**Period_after**	**2**.**188**	**0**.**424**	**5**.**165**	**<0**.**001**
**Status_male_breeder**	**2**.**195**	**0**.**451**	**4**.**865**	**<0**.**001**
**Status_helper**	**2**.**213**	**0**.**664**	**3**.**335**	**<0**.**001**
Days since hatching	0.025	0.043	0.600	0.548
Age_juvenile	−0.128	0.166	−0.769	0.442
Year_1999	−0.022	0.143	−0.152	0.879
**Year_2018**	**−0**.**423**	**0**.**187**	**−2**.**261**	**0**.**024**
Group_control*period_after	**−1**.**367**	**0**.**563**	**−2**.**429**	**0**.**015**
Group_control*status_male	−0.418	0.564	−0.742	0.458
Group_control*status_helper	−1.066	0.905	−1.178	0.239
Status_male_breeder*period_after	**−1**.**672**	**0**.**449**	**−3**.**726**	**<0**.**001**
Status_helper*period_after	**−2**.**171**	**0**.**649**	**−3**.**344**	**<0**.**001**
Group_control*status_male_breeder*period_after	0.945	0.613	1.541	0.123
Group_control*status_helper*period_after	0.719	1.157	0.621	0.535
(b) Prolonging				
Intercept	−0.924	0.533	−1.734	0.083
Group_control	0.192	0.265	0.725	0.468
**Period_after**	**−2**.**002**	**0**.**399**	**−5**.**015**	**<0**.**001**
**Status_male_breeder**	**0**.**790**	**0**.**232**	**3**.**403**	**<0**.**001**
Status_helper	0.225	0.351	0.641	0.521
**Days since hatching**	**0**.**109**	**0**.**050**	**2**.**164**	**0**.**030**
Age_juvenile	−0.163	0.141	−1.157	0.247
Year_1999	0.035	0.118	0.293	0.769
Year_2018	0.041	0.152	0.273	0.784
Group_control*period_after	**1**.**665**	**0**.**470**	**3**.**543**	**<0**.**001**
Group_control*status_male	−0.066	0.319	−0.207	0.836
Group_control*status_helper	−0.54,518	0.636	−0.857	0.391
Status_male_breeder*period_after	**1**.**487**	**0**.**419**	**3**.**546**	**<0**.**001**
Status_helper*period_after	0.955	0.569	1.677	0.093
Group_control*status_male_breeder*period_after	**−1**.**487**	**0**.**530**	**−2**.**807**	**0**.**005**
Group_control*status_helper*period_after	−0.982	0.871	−1.024	0.306

Reference levels for group, period, status, age, and year are “shortened” (a) or “prolonged” (b), “before,” “female,” “adult,” and “1998,” respectively. Significant results (*P* < 0.05) are highlighted in bold. See Methods for details.

**Table 3:** Post hoc contrasts of provisioning rates before and after the manipulation derived from estimated marginal means of the full GLMMs (Table 2), shown separately for (a) the shortening experiment and (b) the prolonging experiment.

(a) Shortening experiment				
Within-status flexibility				
Group	Status	Ratio (±SE)	*Z*	*P*
**Shortened**	**Female breeder**	**8.915** ± **3.776**	**5**.**165**	**<0**.**001**
**Shortened**	**Male breeder**	**1.674** ± **0.290**	**2**.**978**	**0**.**003**
Shortened	Helper	1.017 ± 0.514	0.033	0.974
Control	Female breeder	2.273 ± 0.872	2.139	0.032
Control	Male breeder	1.098 ± 0.220	0.465	0.642
Control	Helper	0.532 ± 0.472	−0.712	0.477

Between-status flexibility				
Group	Contrast	Ratio (± se)	*Z*	*P*
**Shortened**	**Female vs. helper**	**8.768** ± **5.692**	**3**.**344**	**<0**.**001**
**Shortened**	**Female vs. male**	**5.325** ± **2.390**	**3**.**726**	**<0**.**001**
Shortened	Male vs. helper	0.607 ± 0.317	−0.956	0.339
Control	Female vs. helper	4.274 ± 4.098	1.515	0.130
Control	Female vs. male	2.071 ± 0.865	1.742	0.082
Control	Male vs. helper	0.484 ± 0.437	−0.804	0.421

(a) Prolonging experiment				
Within-status flexibility				
Group	Status	Ratio (±SE)	*Z*	*P*
**Prolonged**	**Female breeder**	**0.135** ± **0.054**	**−5**.**015**	**<0**.**001**
**Prolonged**	**Male breeder**	**0.351** ± **0.152**	**−2**.**424**	**0**.**015**
**Prolonged**	**Helper**	**0.597** ± **0.118**	**−2**.**603**	**0**.**009**
Control	Female breeder	0.714 ± 0.203	−1.187	0.235
Control	Male breeder	0.760 ± 0.464	−0.450	0.653
Control	Helper	0.714 ± 0.150	−1.610	0.107

Between-status flexibility				
Group	Contrast	Ratio (± SE)	*Z*	*P*
Prolonged	Female vs. male	0.385 ± 0.219	−1.677	0.094
**Prolonged**	**Female vs. helper**	**0.226** ± **0.095**	**−3**.**546**	**<0**.**001**
Prolonged	Male vs. helper	0.587 ± 0.264	−1.182	0.237
Control	Female vs. male	0.939 ± 0.618	−0.095	0.924
Control	Female vs. helper	1.000 ± 0.324	−0.001	0.999
Control	Male vs. helper	1.065 ± 0.671	0.100	0.920

All contrasts are expressed as after/before ratios on the response scale. For within-status contrasts, values > 1 indicate increased provisioning after the manipulation, whereas values < 1 indicate reduced provisioning. Between-status contrasts compare the magnitude of change (after/before ratios) among carer statuses within each group; in this case, values > 1 indicate a stronger response in the numerator status relative to the denominator status. Significant contrasts (*P* < 0.05) are highlighted in bold.

Between-status post hoc comparisons confirmed marked differences in provisioning flexibility among carer statuses in shortened nests. The increase in provisioning by female breeders was significantly greater than that of helpers (ratio of changes = 8.77 ± 5.692, *P* < 0.001; Table 3, Fig. 3) and male breeders (ratio = 5.325 ± 2.390, *P* < 0.001; Table 3, Fig. 3). In contrast, the magnitude of change did not differ significantly between helpers and male breeders (ratio = 0.607 ± 0.317, *P* = 0.339; Table 3, Fig. 3). No significant differences in flexibility among statuses were detected in control nests (all *P* ≥ 0.08; Table 3, Fig. 3), indicating that the status-specific responses observed in shortened nests were attributable to the experimental manipulation.

### Effect of prolonging on chick provisioning

By contrast, prolonging the chick-rearing period generally reduced provisioning rates (Table 2, Fig. 3). Nevertheless, post hoc contrasts revealed marked status-specific differences in provisioning flexibility. In experimentally prolonged nests, all carer categories significantly reduced their provisioning after receiving older chicks (Table 3, Fig. 3). Female breeders showed the strongest reduction, feeding at rates approximately seven times lower after than before the manipulation (within-status post hoc comparisons: ratio = 0.135 ± 0.054, *P* < 0.001; Table 3, Fig. 3). Male breeders also reduced provisioning, although less strongly (ratio = 0.351 ± 0.152, *P* = 0.015; Table 3, Fig. 3), as did helpers (ratio = 0.597 ± 0.118, *P* = 0.009; Table 3, Fig. 3). In control nests, no significant changes in provisioning were detected for any status (all *P* > 0.10; Table 3, Fig. 3), indicating that the observed reductions in experimental nests were driven by the brood-age manipulation rather than by natural temporal trends.

Between-status post hoc comparisons of provisioning flexibility in experimentally prolonged nests also revealed that female breeders reduced provisioning more strongly than helpers (ratio of changes = 0.226 ± 0.095, *P* < 0.001; Table 3, Fig. 3). Differences between female and male breeders were marginal (ratio = 0.385 ± 0.219, *P* = 0.094, Table 3, Fig. 3), while male breeders did not differ significantly from helpers in their response (ratio = 0.587 ± 0.264, *P* = 0.237; Table 3, Fig. 3). No differences in provisioning flexibility among carer statuses were detected in control nests (all *P* ≥ 0.92; Table 3, Fig. 3), reinforcing that status-specific responses emerged only under experimental brood prolongation.

### Effect of treatment on chick development and body condition before fledging

**Figure 4:**
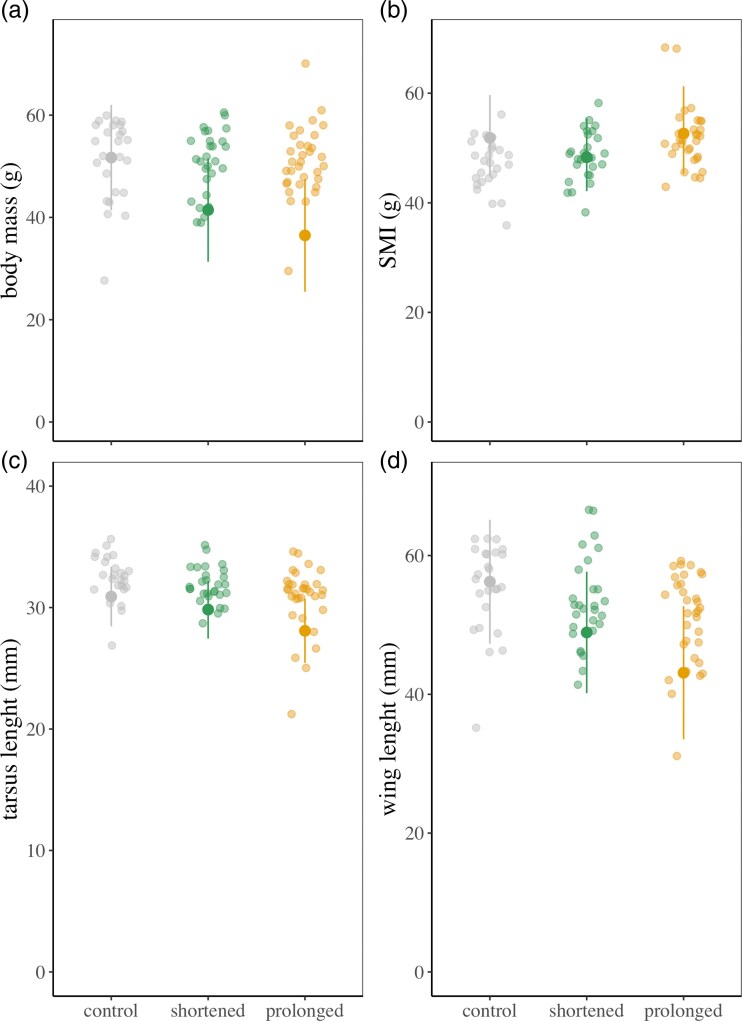
Adjusted predictions for a) body mass, b) scaled mass index (SMI), c) tarsus length, and d) wing length at the control and experimental nests. Predicted values (solid points with 95% CI lines) are based on fixed-effect estimates from the model, adjusted to help = 0, laying date = 19.61, fledglings = 4, year = 1998, and nest = 0 (population-level). Raw data points (jittered) are uncorrected and represent individual observations. Model estimates account for other covariates in the model and may therefore differ from the raw data.

On day 12, chicks from prolonged broods had lower body masses and shorter tarsi and wings than control broods (Fig. 4, Table 4). However, we failed to reveal significant differences in body mass and tarsus length between prolonged and shortened broods (post hoc pair comparisons: body mass: estimate = −4.958 ± 3.406, *Z* = −1.456, *P* = 0.311; tarsus length: estimate = −1.747 ± 0.824, *Z* = −2.121, *P* = 0.085; wing length: estimate = −5.811 ± 2.964, *Z* = −1.961, *P* = 0.121; Fig. 4). Additionally, laying date and the number of nestlings in the nest had a negative effect on body mass but not on tarsus and wing lengths (Fig. 4, Table 4). Finally, body condition did not differ between groups nor was significantly affected by other predictor variables (Fig. 4, Table 4).

**Table 4:** Mixed models explaining variation in chick development and body condition in the treatment groups.

Model	Estimate	SE	df	*T*	*P*
(a) Body mass					
Intercept	71.705	6.259	20.057	11.456	<0.001
**Group_shortened**	**−10**.**253**	**4**.**189**	**13**.**491**	**−2**.**448**	**0**.**029**
**Group_prolonged**	**−15**.**211**	**3**.**995**	**20**.**313**	**−3**.**808**	**0**.**001**
Help	3.564	3.365	11.738	1.059	0.311
**Std. laying date**	**−0**.**558**	**0**.**205**	**12**.**355**	**−2**.**724**	**0**.**018**
No. of **fledglings**	**−2**.**314**	**1**.**045**	**16**.**082**	**−2**.**214**	**0**.**042**
Year_1999	4.8963	4.758	11.681	1.029	0.324
Year_2018	15.025	7.009	11.863	2.144	0.053
(b) SMI					
Intercept	60.520	6.304	28.368	9.601	<0.001
Group_shortened	−4.296	3.905	24.769	−1.100	0.282
Group_prolonged	0.907	4.026	28.196	0.225	0.823
Help	3.177	3.031	23.021	1.048	0.305
Std. laying date	−0.300	0.186	23.094	−1.609	0.121
No. fledglings	−0.328	1.017	27.637	−0.322	0.750
Year_1999	−7.263	4.285	23.171	−1.695	0.104
Year_2018	0.468	6.321	22.942	0.074	0.942
(c) Tarsus length					
Intercept	33.020	1.622	21.750	21.356	<0.001
Group_shortened	−1.072	1.017	17.708	−1.055	0.306
**Group_prolonged**	**−2**.**819**	**1**.**036**	**21**.**687**	**−2**.**721**	**0**.**013**
Help	0.292	0.794	16.064	0.368	0.718
Std. laying date	−0.050	0.049	16.362	−1.017	0.324
No. of fledglings	−0.285	0.263	20.236	−1.086	0.290
Year_1999	2.297	1.122	16.112	2.048	0.057
Year_2018	2.850	1.655	16.070	1.722	0.104
(d) Wing length					
Intercept	67.395	5.530	22.400	12.188	<0.001
Group_shortened	−7.317	3.647	15.634	−2.006	0.062
**Group_prolonged**	**−13**.**127**	**3**.**530**	**22**.**618**	**−3**.**719**	**0**.**001**
Help	−1.521	2.913	13.678	−0.522	0.610
Std. laying date	−0.259	0.178	14.344	−1.456	0.167
No. of fledglings	−1.520	0.916	18.480	−1.658	0.114
Year_1999	3.816	4.118	13.623	0.927	0.370
Year_2018	9.791	6.069	13.807	1.613	0.129

Reference levels for group, help, and year are “control,” “yes,” and “1998,' respectively. Significant results (*P* < 0.05) are highlighted in bold. See Methods for details.

## Discussion

A fundamental question concerning offspring provisioning is the extent to which caregivers adjust their behavior in response to offspring demand. This question remains particularly underexplored in cooperatively breeding systems, where conflicts of interest between helpers and breeders can be pronounced (Trivers 1974; Hatchwell 1999; Clutton-Brock et al. 2004; Koenig and Walters 2012; Khwaja et al. 2017). We examined the provisioning strategies of breeders and helpers in a facultatively cooperative-breeding bird, the Iberian magpie. Our brood manipulation experiment revealed substantial flexibility in chick provisioning. Both breeders and helpers adjusted the duration of parental care: they provisioned chicks in the nest for longer when they received younger foster chicks and for less time when they received older foster chicks. The results also indicate that caregivers are generally flexible, but the magnitude of adjustment varies by status and brood manipulation (shortening vs. prolonging).

In the shortening experiment, both female and male breeders increased provisioning after receiving older chicks, whereas helpers did not. The stronger increase in female provisioning may reflect a shift in parental roles across the nestling period: before the manipulation, females primarily brooded younger chicks and contributed less to feeding than males and helpers (de la Cruz et al. 2019). After receiving older, more demanding chicks, females could reduce brooding and redirect effort toward feeding, resulting in a steeper increase in provisioning rate.

On the other hand, in the prolonging experiment, provisioning generally declined following the manipulation. Breeders (especially females) reduced provisioning when caring for younger chicks for longer, whereas helpers showed weaker and less consistent changes. Between-status comparisons further indicated that female breeders reduced their feeding effort more strongly than helpers, while differences between male breeders and the other carer categories were less pronounced. Together, these results indicate that provisioning responses to brood developmental stage are status-specific, with breeders showing greater flexibility than helpers in both the shortening and prolonging experiments.

Overall, these findings align with previous observational studies showing status-specific provisioning rules in Iberian magpies (Valencia et al. 2003; de la Cruz et al. 2019). Such studies showed that both male and female breeders, but not helpers, increased their parental effort (both visit rate and biomass delivered at each feeding visit) with brood size and brood age—hence the claim “worker males but lazy helpers” (de la Cruz et al. 2019). Several hypotheses have been proposed to explain the optimal amount of investment in birds of different status: the “pay to stay” hypothesis proposes that helpers invest in care as a form of payment to be tolerated within the group (Mulder and Langmore 1993), the “signalling” hypothesis suggests that helping effort advertises individual quality or commitment, potentially yielding future reproductive benefits (Wright 1997), and the “group augmentation” hypothesis posits that helping enhances group size and survival, providing long-term fitness benefits to all members (Kokko et al. 2001). As pointed out by de la Cruz et al. (2019), helping effort in Iberian magpies may act as a signal, irrespective of its intensity, to obtain direct benefits from reciprocity. As in other cooperatively breeding birds, helping effort increases with relatedness, but unrelated helpers still provide substantial care (Wright et al. 2010; de la Cruz et al. 2019). The limited flexibility observed in helpers after receiving older chicks may suggest that helping could be related to a signaling strategy that enhances the recruitment of future helpers (de la Cruz et al. 2019). Alternatively, it may indicate that helpers avoid incurring the additional energetic costs associated with increased care.

While our results are consistent with the flexible-investment hypothesis (Erikstad et al. 2009) and suggest that feeding rules of helpers may be less flexible than those of breeders (Koenig and Walters 2012), it is important to note that provisioning rules in cooperative breeders vary considerably among species. For instance, in obligately cooperative chestnut-crowned babblers (*Pomatostomus ruficeps*), breeders and helpers appear to follow similar provisioning rules (Browning et al. 2012). In meerkats (*Suricata suricatta*, obligately cooperative mongooses), breeders provision offspring at lower rates than helpers and reduce their investment as the number of helpers increases (Clutton-Brock et al. 2004). Such interspecific variation may be partly explained by differences in the likelihood of offspring starvation: when the risk of starvation is high, natural selection is expected to favor carers that maintain their provisioning rates (Hatchwell 1999). A comparative analysis of cooperatively breeding species (Hatchwell 1999) showed that in species where nestling starvation is common, helper contributions are expected to increase overall nest provisioning because breeders do not fully compensate for the presence of helpers. On the other hand, in species where nestlings rarely starve, helpers may or may not increase provisioning. Additionally, variation in provisioning rates may be influenced by differences in the probability of future reproduction between helpers and breeders. Relatively low contributions by breeders might be expected in obligate cooperative breeders, where most helpers never attain reproductive status and the benefits of being a breeder are substantial (Clutton-Brock et al. 2006). In contrast, the contributions of breeders and helpers may be more similar in facultatively cooperative species (Hatchwell 1999).

As expected, none of the experimentally prolonged Iberian magpie broods were deserted, and neither were any of the shortened or control broods. This is in line with previous work showing that Eurasian magpies do not desert after manipulating the parental care period (Soler et al. 2013). As pointed out by Soler et al. (2013), extending the period of parental care may be advantageous as it may compensate for periods of low food intake caused by adverse environmental conditions (eg hot and dry conditions prevailing during the second half of the breeding season; de la Cruz et al. 2022). At the same time, extending parental care may carry costs in terms of future reproductive success; for example, it could potentially delay the onset of a subsequent brood (Rehling et al. 2012). Although second broods are rare in this species (Valencia 2000), replacement clutches are frequent and account for nearly a third of annual breeding success and around a quarter of total nestlings produced in our population (de la Cruz et al. 2022). Therefore, the benefits of prolonged parental care likely outweigh the costs, and natural selection would not favor abandoning broods that are in the nest a few days longer than is typical for their own. Unlike Eurasian magpies, Iberian magpies are not parasitized by brood parasites such as the great spotted cuckoo (*Clamator glandarius*) (Valencia et al. 2005), implying that brood parasitism has not selected for nest desertion when parental care is prolonged. Further, the benefits of continued feeding may increase when aided by helpers, which could buffer against the scarcity of resources during late breeding (de la Cruz et al. 2022). Helping behavior did not affect the duration of the chick-rearing period; yet, this null result may be due to lack of adequate statistical power (only 7 experimental nests and 11 control nests had helpers). This possibility thus requires further investigation.

Our results showed that our brood manipulations had no effect on reproductive success. Interestingly, however, nestlings reared by parents with prolonged parental care period exhibited slower development (lower body masses and shorter tarsi and wings before fledging) than those reared in control broods. Similar results has been found in Eurasian magpies and other bird species, suggesting that the duration of parental care is affected by the state of development of nestlings (Soler et al. 2013). Nonetheless, the interpretation of this result should be treated with caution, especially because we failed to detect significant differences in morphometric measurements between prolonged and shortened broods, and because we did not swap chicks among control broods.

Contrary to our expectations, juveniles and adults showed similar provisioning rates, irrespective of brood size and nestling age, suggesting no age-specific differences in provisioning effort. This is partly consistent with observations in chestnut-crowned babblers, where helpers exhibited similar levels of effort regardless of their age (Browning et al. 2012). We acknowledge that relying solely on delivery rate may result in underestimating or overestimating the actual food contribution to the nest, so we cannot rule out the possibility that birds adjusted the type of prey and/or biomass delivered depending on their age. In fact, Iberian magpie breeders increase their feeding rate and the biomass delivered as the brood demands increase (de la Cruz et al. 2019). Furthermore, provisioning effort is likely to increase with relatedness (Nam et al. 2010; de la Cruz et al. 2019). Indeed, a previous observational study on the same population found that first option helpers increased their feeding rate as the brood aged, whereas second option helpers maintained their parental effort (de la Cruz et al. 2019). Unfortunately, we lack sufficient statistical power to make comparisons between first and second option helpers due to low sample sizes. To complicate the matter more, provisioning behavior could also be influenced by the sex of offspring (Lessells 2002). Sex-biased investment has been demonstrated in several cooperatively breeding species, and the provisioning effort of breeders and helpers is often biased toward the more valuable sex (Nam et al. 2011). According to the “local resource enhancement” hypothesis, Iberian magpie breeders and helpers should preferentially invest in male chicks that will remain in their natal colony and are likely to reciprocate in future (de la Cruz and Valencia 2016). Yet, philopatric males may compete with parents and helpers for access to resources such as food or mates (“local resource competition” hypothesis; Clark 1978; Gowaty 1993). Thus, future studies should also consider the amount of food biomass delivered by breeders and helpers as well as their relatedness to the helped brood and the sex of offspring that they provision to determine fine-scale adjustments in provisioning effort.

To summarize, this study adds to evidence that the flexible-investment hypothesis applies to cooperative breeding systems. Our findings show that both breeders and helpers adjust the duration of the nestling care period to offspring requirement and that breeders, overall, adjusted their provisioning rates more strongly than helpers. This pattern supports the notion that feeding rules of helpers are less flexible than those of breeders (Koenig and Walters 2012).

## Supplementary Material

arag021_Supplementary_Data

## Data Availability

Analyses reported in this article can be reproduced using the data provided by Gutiérrez et al. (2026).
